# Experimental datasets on processed eggshell membrane powder for wound healing

**DOI:** 10.1016/j.dib.2019.104457

**Published:** 2019-08-31

**Authors:** Tamer A.E. Ahmed, Henri-Pierre Suso, Maxwell T. Hincke

**Affiliations:** a Medical Biotechnology Department, Genetic Engineering and Biotechnology Research Institute, City of Scientific Research and Technology Applications (SRTA-City), Alexandria, Egypt; b Department of Cellular and Molecular Medicine, Faculty of Medicine, University of Ottawa, Ottawa, Ontario, K1H 8M5, Canada; c Biovotec AS, Engebrets Vei 3, 0275, Oslo, Norway; d Department of Innovation in Medical Education, Faculty of Medicine, University of Ottawa, Ottawa, Ontario, K1G 5Z3, Canada

**Keywords:** Processed eggshell membrane powder, Proteomics, Wound healing, Histological scoring

## Abstract

Eggshell (ES) and eggshell membrane (ESM) is a significant byproduct of the egg producing industry (Ahmed et al., 2019). Many studies have been undertaken to utilize ES waste for potential value added applications (Cordeiro and Hincke, 2011). Described here are the datasets from our evaluation of processed eggshell membrane powder (PEP) as a wound healing product using the mouse excisional wound splinting model (Ahmed et al., 2019). PEP biomaterial was characterized by proteomics using various extraction and solubilization strategies including moderate (lithium dodecyl sulphate (LDS) and urea/ammonium bicarbonate) and harsh conditions (3-mercaptopropionic acid (3-MPA) and NaOH/dimethylsulfoxide) in order to progressively overcome its stable, insoluble nature (Ahmed et al., 2019, Ahmed et al., 2017). Analysis of proteomic data allowed the relative abundance of the main PEP protein constituents to be determined. The efficacy of PEP for promotion of wound healing was assessed using the mouse excisional wound splinting model, and well-established semi-quantitative histological scoring. (More details about the PEP biomaterial characterization and its *in vivo* evaluation can be found in the related research article (Ahmed et al., 2019)).

**Table:** Specifications Table

Subject area	*Materials Science*
More specific subject area	*Biomaterials*
Type of data	*Tables and figures.*
How data was acquired	*Agilent 1200 nanopump (Reversed-phase (RP) nanoscale capillary liquid chromatography (nanoLC), Agilent Technologies Canada Inc., Ontario, Canada) connected to mass spectrometer 5600 with a nanoelectrospray ion source (ES-MS/MS, AB Sciex, MA, USA).* *LOGOS microwave hybrid tissue processor (Milestone, MI, USA).* *Leica microtome (Leica Biosystems Inc., ON, Canada).* *Zeiss Mirax Midi whole slide digital scanner (Carl Zeiss Canada Ltd, ON, Canada).*
Data format	*Raw and analyzed*
Experimental factors	***Mass spectrometry****: Eggshell membrane collected at the egg breaking unit was processed (washed, milled, sieved, and γ sterilized) into a micronized powder (<100 μm), which was designated “Processed Eggshell Membrane Powder” (PEP). For proteomics, PEP samples were subjected to various extraction and solubilization strategies including moderate (via lithium dodecyl sulphate (LDS) and urea/ammonium bicarbonate (NH*_*4*_*HCO*_*3*_*)) and harsh conditions (via 3-mercaptopropionic acid (3-MPA) and NaOH/dimethylsulfoxide) conditions. Samples prepared by 3-MPA, NaOH/DMSO, and LDS/DTT treatment were subjected to in-gel digestion, while in the case of urea/NH*_*4*_*HCO*_*3*_*extraction, in-solution digestion was performed. The protein constituents of PEP were identified using LC/MS/MS analysis, with a false discovery rate (FDR) of 1% and at least two unique peptides. Keratins were discarded from the identified protein inventory. In addition, any protein identified with only one unique peptide (according to the Scaffold software interface) was discarded from the final protein inventory.* ***Tissue processing****: The processed wound samples were cut into two halves (Upper and lower halves) and then embedded in paraffin (Leica Biosystems Inc., ON, Canada). PEP (50 mg) was suspended in PBS and centrifuged. The resultant pellet was centered in pre-embedding media and processed with the LOGOS tissue processor.* ***Digital scanning:****Stained tissue and PEP sections were scanned with Zeiss Mirax Midi whole slide digital scanner (12 slides/scan and 40X objective lens). Exposure time was 10-100 ms (bright field) and the specimen threshold level of 40-45.*
Experimental features	*A complete protein inventory for PEP was created by merging the proteins identified by LC/MS/MS analysis after various extraction and solubilization strategies. Relative abundances of proteins identified in the PEP biomaterial were determined using Scaffold proteome software. The effect of PEP on wound healing was evaluated in the mouse excisional wound splinting model using the macroscopic planimetric timecourse (30–38 mice) and a histological scoring system (4 mice each at time points 3, 10, and 17). Various histological parameters related to wound healing were scored for all stained section. The absence of stainable collagen in the PEP biomaterial was confirmed using Masson's trichrome staining of the PEP pellet.*
Data source location	*MS/MS spectrometry was conducted in the Proteomics Platform Of Québec Genomics Center, CHU de Québec Research Center (Laval, QC, Canada).* *In vivo experiments were carried out in the animal care and veterinary service facility (ACVS), Faculty of Medicine, University of Ottawa, Ottawa, ON, Canada.* *Wound tissue sample processing, embedding, sectioning, staining, and scanning was performed in the Histology Core Facility, Department of Pathology and Laboratory Medicine, Faculty of Medicine, University of Ottawa, Ottawa, ON, Canada.*
Data accessibility	*The data are available within the article.*
Related research article	*Ahmed TAE, Suso HP, Maqbool A, and Hincke MT. Processed Eggshell Membrane Powder: Bioinspiration for an Innovative Wound Healing Product, Mater Sci Eng C Mater Biol Appl. 95 (2019) 192–203.*

**Table:** 

**Value of the data** • The presented data describes the utilization of various extraction and solubilization strategies [1], [4] to identify the protein constituents of PEP by proteomics. • The proteomic approach allows the estimation of relative abundances of the main protein constituents of PEP biomaterial. • The data demonstrates the use of animals (C57BL/6J mice) for the planimetric timecourse and histological assessment of healing of the splinted excisional wound after application of a biomaterial (PEP). • The data describes an established histological scoring system used to assess the effect of PEP on various histological parameters critical to assess wound healing promotion. • The histological processing of PEP biomaterial via pelleting and pre-embedding in agar-formalin media provides researchers with a strategy to process powdered biomaterials and even cells.

## 1 Data

**Fig. 1:**
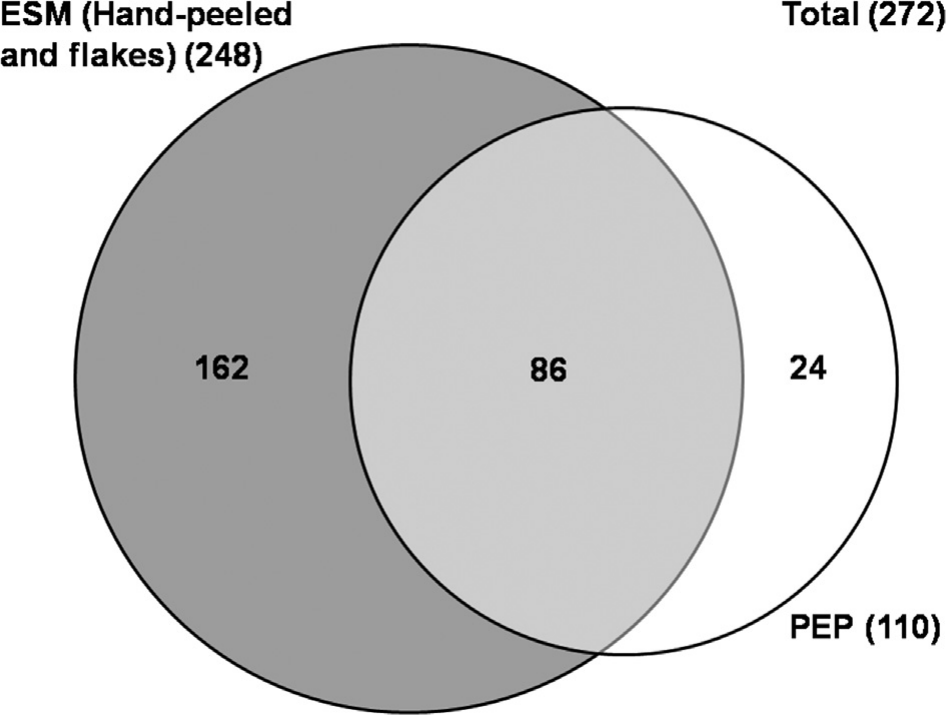
Venn chart showing a comparison of the PEP proteome to the recently published ESM proteome (Ahmed et al., 2017) [4]. Twenty four (24) of the PEP proteins were not previously identified in the ESM proteome. Micronization to prepare PEP facilitated the identification of a greater number of proteins by increasing the efficiency of the in-solution digestion approach.

**Fig. 2:**
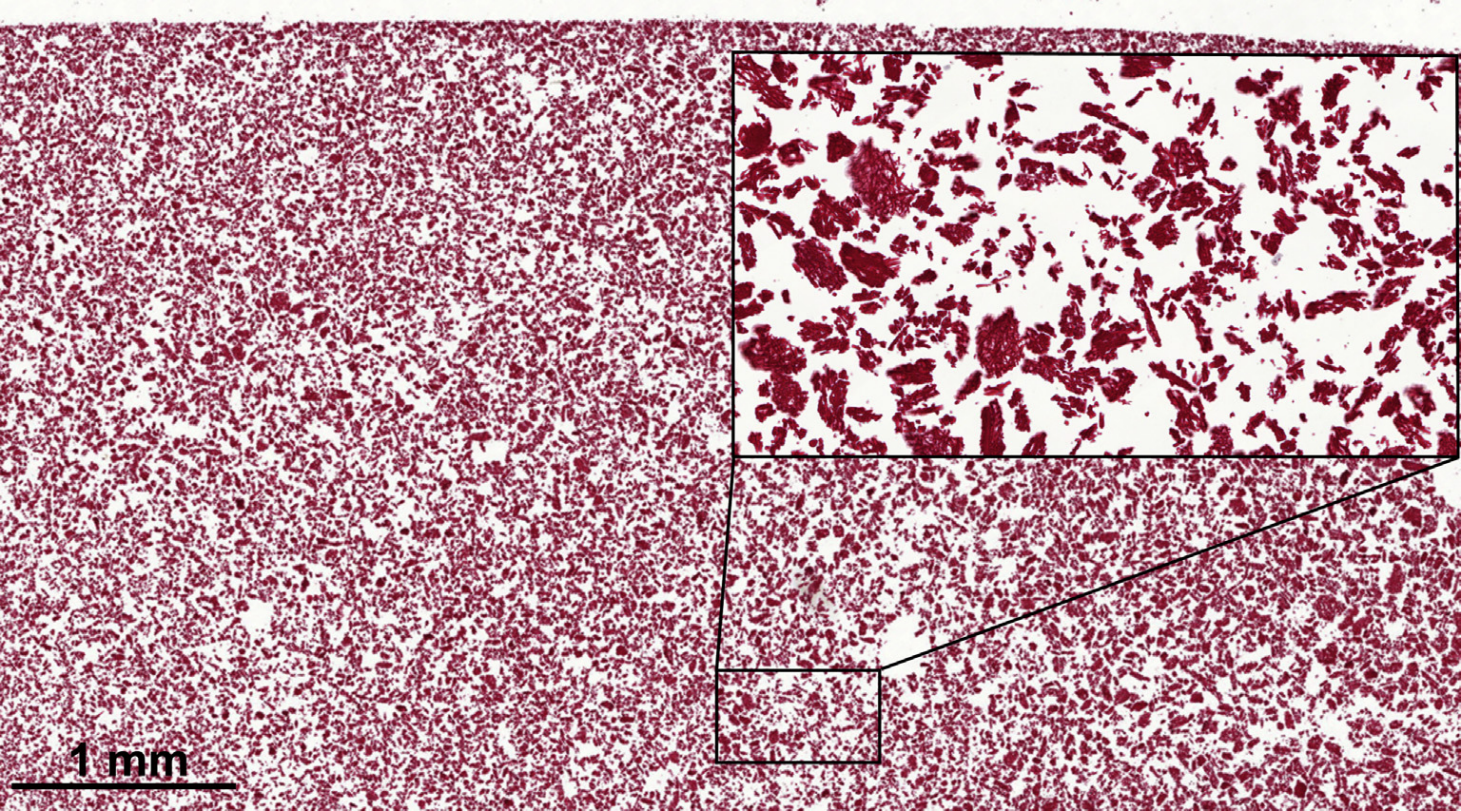
Masson's trichrome staining showing the amorphous nature of PEP biomaterial and the absence of stainable collagen fibres.

The presented data demonstrates the utilization of various extraction strategies (moderate to harsh conditions) [1], [2], [3], [4] to identify the protein constituents of PEP using the proteomic approach (Table 1). A comprehensive PEP proteome was established and compared to the general ESM proteome (Fig. 1 and Table 2). LC/MS/MS spectrometry data was interpreted in order to determine the relative abundance of the main protein constituents of PEP biomaterial (Table 3). The kinetics of wound healing (with and without PEP) in the mouse splinting excisional wound model was determined using a macroscopic planimetric strategy with histological scoring (Table 4). The histological scoring system was established to assess various histological parameters including degree of angiogenesis, collagen deposition, fibroblast infiltration, macrophage infiltration, polymorphonuclear cells (PMN) infiltration, fibrin clot formation, epidermal differentiation and indentation along with the presence of multinucleated giant cells (Table 5). Finally, PEP was stained with Masson's trichrome to confirm the absence of stainable collagen using an innovative pre-embedding histological approach (Fig. 2).

**Table 1:** Various extraction conditions used for the in-solution and in-gel digestion-based proteomic analysis of PEP.

Extraction strategy			
In-solution digestion^a^	In-gel digestion		
	A^b^	B^b^	C^a^
Digestion buffer (urea 8 M/ammonium bicarbonate 100 mM), sonication (2 × 15s on – 1min off on ice), centrifugation (16,000×g, 10min, 4 °C)	3-mercaptopropionic acid (1.25 M), 1.7 M acetic acid, 24 hours, 80 °C, shaking water bath.	NaOH (5% w/v), DMSO, 4 hours, 50 °C, hot plate stirrer.	LDS (73mM)/DTT (50 mM), NuPAGE sample buffer only, 30 minutes, 70 °C, Heat block.

a Moderate extraction conditions.

b Harsh solubilization conditions.

**Table 2:** Inventory of the PEP proteome, as compared to the ESM (hand-peeled and flakes) proteome.

No.	Protein name	Gene Symbol	Gene ID	PEP	ESM
1	Actin, γ1	ACTG1	415296	YES	YES
2	ADAM metallopeptidase with thrombospondin type 1 motif, 5	ADAMTS5	427971	YES	NO
3	A-kinase anchoring protein 12	AKAP12	421634	NO	YES
4	Albumin	ALB	396197	YES	YES
5	Aminopeptidase N, Alanyl (membrane) aminopeptidase.	ANPEP	395667	NO	YES
6	Angiopoietin like 3	ANGPTL3	100189558	YES	NO
7	Annexin A2	ANXA2	396297	YES	NO
8	Antigen identified by monoclonal antibody Ki-67	MKI67	423963	NO	YES
9	Apolipoprotein A-I	APOA1	396536	YES	YES
10	Apolipoprotein B	APOB	396535	YES	YES
11	Apolipoprotein D	APOD	424893	YES	YES
12	Apolipoprotein H (β-2-glycoprotein I)	APOH	417431	YES	YES
13	Apovitellenin 1	APOV1	396476	YES	YES
14	ash1 (absent, small, or homeotic)-like	ASH1L	425064	NO	YES
15	ATPase H+ transporting accessory protein 2	ATP6AP2	418573	YES	NO
16	ATP-binding cassette, sub-family A (ABC1), member 4	ABCA4	424490	NO	YES
17	Avian β-defensin 9	AvBD9	414343	NO	YES
18	Avian β -defensin 10	AvBD10	414341	NO	YES
19	Avian β -defensin 11	AvBD11	414876	YES	YES
20	Avidin	AVD	396260	YES	YES
21	BPI fold containing family C, member B	BPIFCB	771461	NO	YES
22	Breast cancer 2	BRCA2	374139	NO	YES
23	Bromodomain containing 8	BRD8	416219	NO	YES
24	BTB domain containing 7	BTBD7	423424	NO	YES
25	Ca++-dependent secretion activator 2	CADPS2	417756	NO	YES
26	Cadherin 1, type 1, E-cadherin (epithelial)	CDH1	415860	YES	YES
27	Cadherin, EGF LAG seven-pass G-type receptor 3	CELSR3	107054381	NO	YES
28	Calcium channel, voltage-dependent, T type, a 1H subunit	CACNA1H	416526	NO	YES
29	Calcium/calmodulin-dependent protein kinase II β	CAMK2B	374174	NO	YES
30	Calmodulin 2	CALM	395855	NO	YES
31	Carbohydrate (N-acetylglucosamine 6-O) sulfotransferase 6	CHST6	770257	YES	NO
32	Carboxypeptidase E	CPE	422424	YES	NO
33	Cathepsin B	CTSB	396329	YES	YES
34	Cathepsin E-A-like	CTSEAL	417848	YES	NO
35	Cell division cycle 20B	CDC20B	426169	NO	YES
36	Centriolin	CNTRL	417121	NO	YES
37	Centrosomal protein 152kDa	CEP152	415437	NO	YES
38	Chondroitin sulphate proteoglycan 4	CSPG4	425524	NO	YES
39	Chromosome 1 open reading frame, human C12orf35	C1H12ORF35	418136	NO	YES
40	Clusterin	CLU	395722	YES	YES
41	Coagulation factor II (thrombin)	F2	395306	NO	YES
42	Cochlin	COCH	395779	NO	YES
43	Collagen III (α1 chain)	COL3A1	396340	NO	YES
44	Collagen IV (α 1 chain)	COL4A1	395530	NO	YES
45	Collagen IV (α 3 chain)	COL4A3	424797	NO	YES
46	Collagen IV (α 6 chain)	COL4A6	422350	NO	YES
47	Collagen V (α 2 chain)	COL5A2	423986	NO	YES
48	Collagen VII (α 1 chain)	COL7A1	427584	NO	YES
49	Collagen VIII (α 1 chain)	COL8A1	418378	NO	YES
50	Collagen X (α 1 chain)	COL10A1	100858979	YES	YES
51	Collagen XI (al chain)	COL11A1	374046	NO	YES
52	Collagen XII (al chain)	COL12A1	395875	NO	YES
53	Collagen XXII, (a l chain)	COL22A1	420315	NO	YES
54	Complement component 3	C3	396370	YES	NO
55	Contactin 5	CNTN5	395317	NO	YES
56	Cortactin binding protein 2	CTTNBP2	417766	NO	YES
57	Corticotropin releasing hormone	CRH	404297	NO	YES
58	CREMP (cysteine rich ESM protein)	CREMP	776923	YES	YES
59	CREMP1	N/A	N/A	NO	YES
60	CREMP2	N/A	N/A	NO	YES
61	CREMP3	N/A	N/A	YES	YES
62	CREMP4	N/A	N/A	NO	YES
63	CREMP5	N/A	N/A	YES	YES
64	CREMP6	N/A	N/A	NO	YES
65	CTS telomere maintenance complex component 1	CTC1	418324	NO	YES
66	CUB and Sushi multiple domains 2	CSMD2	419640	NO	YES
67	Cystatin C	CST3	396497	YES	YES
68	Dedicator of cytokinesis 1	DOCK1	423960	NO	YES
69	Deleted in malignant brain tumors 1 protein-like (EW135).	DMBT1L	426826	YES	YES
70	DENN/MADD domain containing 4C	DENND4C	427236	NO	YES
71	Desmoplakin	DSP	420869	NO	YES
72	Dickkopf homolog 3	DKK3	396023	YES	YES
73	di-N-acetyl- chitobiase	CTBS	424535	NO	YES
74	DnaJ heat shock protein family (Hsp40) member C7	DNAJC7	428312	NO	YES
75	Dynein, axonemal, heavy chain 1	DNAH1	415943	NO	YES
76	Dynein, axonemal, heavy chain 12	DNAH12	416004	NO	YES
77	Dynein, axonemal, heavy chain 9	DNAH9	417314	NO	YES
78	Dynein, cytoplasmic 2, heavy chain 1	DYNC2H1	418979	NO	YES
79	Dystrophin	DMD	396236	NO	YES
80	EGF containing fibulin-like extracellular matrix protein 1	EFEMP1	428543	NO	YES
81	EGF-like repeats and discoidin I-like domains 3	EDIL3	427326	YES	YES
82	Enolase 2 (γ, neuronal)	ENO2	395689	NO	YES
83	Enolase 3	ENO3	396016	NO	YES
84	EPH receptor B3	EPHB3	396179	NO	YES
85	Eukaryotic translation elongation factor l al	EEF1A1	373963	NO	YES
86	Family with sequence similarity 20, member C	FAM20C	416445	YES	NO
87	Family with sequence similarity 21, member A	FAM21A	423772	NO	YES
88	F-box and WD repeat domain containing 8	FBXW8	417024	NO	YES
89	Fibrinogen γ chain	FGG	395837	YES	NO
90	Fibronectin 1	FN1	396133	YES	YES
91	flightless I homolog	FLII	416515	NO	YES
92	Folate receptor 1 (adult)	FOLR1	395638	NO	YES
93	G protein-coupled receptor kinase interactor 1	GIT1	417584	NO	YES
94	G protein-coupled receptor kinase interactor 2	GIT2	374035	NO	YES
95	Galactosylceramidase	GLAC	423394	YES	YES
96	Gastrokine 2	GKN2	419515	YES	YES
97	Glutamine and serine rich 1	QSER1	421599	NO	YES
98	Glutathione peroxidase 3	GPX3	427638	YES	YES
99	Glutathione S-transferase α 3	GSTA3	414896	NO	YES
100	Golgi glycoprotein 1	GLG1	396492	YES	NO
101	Group-specific component (vitamin D binding protein)	GC	395696	NO	YES
102	Heat shock 70kDa protein 5 (glucose-regulated protein, 78kDa)	HSPA5	396487	YES	NO
103	Heat shock 70kDa protein 8	HSPA8	395853	NO	YES
104	Hemoglobin, α 1	HBAA	416652	YES	NO
105	Hemoglobin, γ G	HBG2	396485	YES	YES
106	Hemopexin	HPX	419076	YES	YES
107	HEP21 protein	HEP21	395192	YES	YES
108	Heterogeneous nuclear ribonucleoprotein A2/B1	HNRNPA2B1	420627	NO	YES
109	Heterogeneous nuclear ribonucleoprotein A3 homolog 1 -like	HNRNPA3	100859627	NO	YES
110	Heterogeneous nuclear ribonucleoprotein D-like	HNRNPDL	422601	NO	YES
111	Hexosaminidase B (β polypeptide)	HEXB	427204	YES	NO
112	Histone H1.11L	HIST1H111L	427892	NO	YES
113	Histone H1.11R	HIST1H111R	427896	NO	YES
114	Histone H2A	HIST1H2A4	404299	NO	YES
115	Histone H2B	HIST1H2B8	427886	YES	YES
116	Histone H3 family 3C	H3F3C	427887	NO	YES
117	Histone H4	HIST1H47	417950	YES	YES
118	Histone H5	H1F0	693250	NO	YES
119	Hyaluronan and proteoglycan link protein 3	HAPLN3	415495	YES	YES
120	Ig heavy chain	N/A	N/A	NO	YES
121	Ig heavy chain variable region	N/A	N/A	NO	YES
122	Ig J polypeptide, linker protein for Ig a and α polypeptides	IGJ	374117	NO	YES
123	Ig light chain variable region	N/A	N/A	NO	YES
124	Ig mu chain C region	N/A	N/A	YES	YES
125	Ig α heavy chain	N/A	N/A	YES	YES
126	Ig γchain	N/A	N/A	YES	YES
127	Ig λlight chain	N/A	N/A	NO	YES
128	Ig λ-like polypeptide 1	IGLL1	416928	YES	YES
129	Immunoglobulin like domain containing receptor 1	ILDR1	418358	NO	YES
130	Junction plakoglobin	JUP	429710	NO	YES
131	Kinesin family member 21B	KIF21B	421178	NO	YES
132	Kinesin family member 26A	KIF26A	423489	NO	YES
133	La ribonucleoprotein domain family, member 4B	LARP4B	420457	NO	YES
134	LDL receptor-related protein 11	LRP11	421629	NO	YES
135	Lectin, mannose-binding 2	LMAN2	100859676	NO	YES
136	Leucine zipper protein 1	LUZP1	428210	NO	YES
137	Lipocalin 8, extracellular fatty acid-binding protein	LCN	396393	YES	YES
138	Lymphocyte antigen 86	LY86	420872	YES	YES
139	Lysozyme C	LYZ	396218	YES	YES
140	Lysyl oxidase-like 2	LOXL2	419533	YES	YES
141	Mediator complex subunit 15	MED15	416941	NO	YES
142	Melanoma inhibitory activity family, member 3	MIA3	421337	NO	YES
143	Milk fat globule-EGF factor 8 protein (lactadherin isoform 2)	MFGE8	415494	YES	YES
144	Mucin 6 oligomeric mucus/gel-forming (ovomucin, β subunint)	MUC6	414878	YES	YES
145	Mucin-5AC-like	LOC100859916	100859916	YES	YES
146	Myeloid/lymphoid or mixed-lineage leukemia 2	MLL2	425846	NO	YES
147	Myeloid/lymphoid or mixed-lineage leukemia 3	MLL3	420437	NO	YES
148	Myosin, heavy chain 10, non-muscle	MYH10	396465	NO	YES
149	Myosin, heavy chain 9, non-muscle	MYH9	396469	NO	YES
150	N-acetylglucosamine-1-phosphate transferase, a and β subunits	GNPTAB	418096	NO	YES
151	Neuron navigator 2	NAV2	422977	NO	YES
152	Neuron navigator 3	NAV3	417869	NO	YES
153	Neuropeptide Y	NPY	396464	NO	YES
154	Neurotrimin	NTM	395450	NO	YES
155	Nucleobindin 2	NUCB2	423071	YES	YES
156	Obscurin, cytoskeletal calmodulin and titin-interacting RhoGEF	OBSCN	420395	NO	YES
157	Olfactomedin 4, tiarin-like	OLFM4	418826	YES	YES
158	Ovalbumin	SERBIN14	396058	YES	YES
159	Ovalbumin-related protein X	SERPINB14C	420898	YES	YES
160	Ovalbumin-related protein Y	SERPINB14B	420897	YES	YES
161	Ovocalyxin 32 (Retinoic acid receptor responder 1)	RARRES1	395209	YES	YES
162	Ovocalyxin 36 (BPI fold containing family B, member 3)	BPIFB3	419289	YES	YES
163	Ovocleidin 116 (matrix extracellular phosphoglycoprotein)	MEPE	395256	YES	YES
164	Ovocleidin 17	OC-17	100313508	YES	YES
165	Ovoglobulin G2 (TENP)	BPIFB7	395882	YES	YES
166	Ovodefensin A1	OvoDA1	422030	YES	YES
167	Ovomucin, α subunit	MUC5B	395381	YES	YES
168	Ovostatin	OVST	396151	YES	YES
169	Ovostatin-like	OVSTL	425757	NO	YES
170	Ovotransferrin (transferrin)	TF	396241	YES	YES
171	p21 protein (Cdc42/Rac)-activated kinase 3	PAK3	422342	NO	YES
172	Phosphoglucomutase 5	PGM5	427215	NO	YES
173	Phospholipase B domain containing 1	PLBD1	417967	YES	NO
174	Piccolo (presynaptic cytomatrix protein)	PCLO	395319	NO	YES
175	PIT54 protein	PIT54	395364	YES	YES
176	Pleiotrophin	PTN	418125	YES	NO
177	Polycystic kidney and hepatic disease 1 (autosomal recessive)	PKHD1	422044	NO	YES
178	Polymeric immunoglobulin receptor	PIGR	419848	NO	YES
179	Procollagen-lysine, 2-oxoglutarate 5-dioxygenase 1	PLOD1	419485	YES	YES
180	Programmed cell death 6	PDCD6	420988	NO	YES
181	Prolyl 4-hydroxylase, β polypeptide	P4HB	374091	YES	NO
182	Prospero homeobox 1	PROX1	395802	NO	YES
183	Prostaglandin D2 synthase 21kDa	PTGDS	374110	YES	YES
184	Prostate stem cell antigen	PSCA	420302	YES	YES
185	Prostatic acid phosphatase-like	LOC428451	428451	YES	YES
186	Protein O-fucosyltransferase 2	POFUT2	395112	YES	NO
187	Protein phosphatase, Mg2+/Mn2+ dependent, 1J	PPM1J	419873	NO	YES
188	Protein tyrosine phosphatase, receptor type, A	PTPRA	396060	NO	YES
189	Protocadherin 1	PCDH1	416194	NO	YES
190	Quiescin Q6 sulfhydryl oxidase 1	QSOX1	373914	YES	YES
191	Retbindin (Riboflavin-binding protein)	RTBDN	396449	YES	YES
192	Retinoic acid receptor responder 2	RARRES2	420366	YES	YES
193	Rho guanine nucleotide exchange factor (GEF) 17	ARHGEF17	777518	NO	YES
194	Ribosomal protein L36	RPL36	373936	NO	YES
195	Ring finger protein 17	RNF17	418961	NO	YES
196	Rootletin, ciliary rootlet coiled-coil	CROCC	428191	NO	YES
197	RPE-spondin-like	LOC771089	771089	NO	YES
198	Salivary amylase, αlA	AMY1A	414139	NO	YES
199	Sal-like 4	SALL4	769286	NO	YES
200	Secretoglobin family 1C member 1 -like	LOC101749303	101749303	NO	YES
201	Secretory trypsin inhibitor	SPINK1	101749216	NO	YES
202	Sema domain, immunoglobulin domain (Ig), short basic domain, secreted, (semaphorin) 3G	SEMA3G	415945	YES	YES
203	Serine peptidase inhibitor, Kazal type 2 (acrosin-trypsin inhibitor)	SPINK2	770729	YES	YES
204	Serine peptidase inhibitor, Kazal type 5, (Ovoinhibitor)	SPINK5	416235	YES	YES
205	Serine peptidase inhibitor, Kazal type 7 (ovomucoid)	SPINK7	416236	YES	YES
206	Serine/threonine kinase 38	STK38	428260	NO	YES
207	Serpin peptidase inhibitor, clade B (ovalbumin), member 1	SERPINB1	420894	NO	YES
208	Serpin peptidase inhibitor, clade B (ovalbumin), member 5	SERPINB5	420900	NO	YES
209	Serpin peptidase inhibitor, clade E (nexin, plasminogen activator inhibitor type 1), member 2	SERPINE2	424805	YES	YES
210	Serpin peptidase inhibitor, clade F (α-2 antiplasmin, pigment epithelium derived factor), member 2	SERPINF2	100857105	YES	YES
211	Shroom family member 3	SHROOM3	422636	NO	YES
212	Similar to arf-GAP with Rho-GAP domain of Zebrafish	N/A	N/A	NO	YES
213	Similar to CREB binding protein b of Zebrafish	N/A	N/A	NO	YES
214	Similar to cadherin 4 of Zebrafish	CDH4	N/A	NO	YES
215	Similar to Calumenin A of Zebrafish	N/A	N/A	YES	YES
216	Similar to IgGFc-binding protein-like of wild turkey.	ZAN	N/A	NO	YES
217	Similar to Kunitz-like protease inhibitor	LOC771972	771972	YES	YES
218	Similar to metastasis associated 1 of Zebrafish	MTA1	N/A	NO	YES
219	Similar to Septin 4a of Zebrafish	N/A	N/A	NO	YES
220	Similar to transcription factor EB Zebrafish	TFEB	N/A	NO	YES
221	Similar to zinc finger ZZ-type and EF-hand domain-containing protein 1 of wild turkey	ZZEF1	100541118	NO	YES
222	Spectrin repeat containing, nuclear envelope 1	SYNE1	421640	YES	YES
223	Spectrin, β, non-erythrocytic 5	SPTBN5	423225	NO	YES
224	Sperm associated antigen 16	SPAG16	424009	NO	YES
225	Stromal cell derived factor	SDF4	419423	YES	YES
226	Syndecan binding protein (syntenin)	SDCBP	421136	YES	NO
227	TATA box binding protein like	TBPL2	776269	NO	YES
228	Tenascin C	TNC	396440	YES	YES
229	Teneurin transmembrane protein 3	TENM3	422557	NO	YES
230	Tetratricopeptide repeat domain 3	TTC3	418518	NO	YES
231	Thyroid hormone receptor interactor 11	TRIP11	423414	NO	YES
232	TIMP metallopeptidase inhibitor 3	TIMP3	396483	YES	YES
233	Titin	TTN	424126	NO	YES
234	transcobalamin 2	TCN2	429737	YES	NO
235	Transient receptor potential cation channel, subfamily M, member 1	TRPM1	427494	NO	YES
236	Transient receptor potential cation channel, subfamily V, member 2	TRPV2	417603	NO	YES
237	Transthyretin.	TTR	396277	YES	YES
238	Tsukushi, small leucine rich proteoglycan	TSKU	419088	YES	YES
239	Tumor necrosis factor receptor superfamily, member 6b, decoy	TNFRSF6B	395096	YES	YES
240	Tumor necrosis factor superfamily member 10	TNFSF10	378894	YES	NO
241	Ubiquitin B	UBB	396190	NO	YES
242	Ubiquitin specific peptidase 4 (proto-oncogene)	USP4	415937	NO	YES
243	Ubiquitin-protein ligase E3B	UBE3B	776286	NO	YES
244	Uncharacterized LOC107049386	LOC107049386	107049386	NO	YES
245	Uncharacterized LOC771994	LOC771994	771994	YES	NO
246	Uncharacterized protein (R4GJG8)	N/A	N/A	NO	YES
247	Uncharacterized protein (UPI0000448E55)	N/A	N/A	YES	NO
248	Uncharacterized protein (UPI0000E802A1)	N/A	N/A	YES	NO
249	Uncharacterized protein (UPI000240B987)	N/A	N/A	NO	YES
250	Uncharacterized proteins (R4GIK1)	N/A	N/A	NO	YES
251	Uridine-cytidine kinase 1 -like 1	UCKL1	419255	NO	YES
252	Vacuolar protein sorting 13 homolog D	VPS13D	419481	NO	YES
253	Vitelline membrane outer layer protein 1	VMO1	418974	YES	YES
254	Vitellogenin 1	VTG1	424547	YES	YES
255	Vitellogenin 2	VTG2	424533	YES	YES
256	Vitronectin	VTN	395935	YES	YES
257	v-raf murine sarcoma viral oncogene homolog B	BRAF	396239	NO	YES
258	WAP four-disulfide core domain 8	WFDC8	419301	YES	YES
259	WSC domain containing 2	WSCD2	416887	NO	YES
260	YLP motif containing 1; (C14orf170)	YLPM1	423356	NO	YES
261	Zinc finger protein 185-like	LOC422301	422301	NO	YES
262	Zinc finger protein 335	ZNF335	396131	NO	YES
263	Zinc finger, CCHC domain containing 11	ZCCHC11	424642	NO	YES
264	Zona pellucida glycoprotein 1 (sperm receptor)	ZP1	395418	NO	YES
265	Zona pellucida sperm-binding protein 3	ZP3	378906	NO	YES
266	A thalassemia/mental retardation syndrome X-linked	ATRX	422331	NO	YES
267	α1 acid glycoprotein	ORM1	395220	YES	YES
268	α2 macroglobulin-like 1	A2ML1	418254	YES	YES
269	α2 macroglobulin-like 4	A2ML4	100858010	NO	YES
270	β 1,4-N-acetyl-galactosaminyl transferase 4	B4GALNT	4770601	NO	YES
271	β microseminoprotein-like	LOC101750704	101750704	YES	YES
272	β2 microglobulin	B2M	414830	NO	YES
Total				110	248

**Table 3:** Relative abundance of the main proteins constituting the PEP biomaterial. Data is arranged according to the percent abundance.

Gene symbol	Average total spectral count	% abundance
LOXL2	33.3	28.0
CREMPs	31.2	27.0
LYZ	13.8	12.0
COL10A1	11.5	10.0
SERBIN14	7.3	6.0
MEPE	4.0	3.0
TF	3.0	3.0
CLU	2.0	2.0
HAPLN3	2.0	2.0
OC-17	2.5	2.0
GKN2	1.0	0.8
NUCB2	1.0	0.8
ORM1	1.0	0.8
QSOX1	1.0	0.8
SERPINB14B	1.0	0.8
SERPINB14C	1.0	0.8
VTG2	1.0	0.8

**Table 4:** Number of mice used for the *in vivo* study.

Purpose of the study	Number of C57BL/6J mice evaluated					
	Day 0	Day 3	Day 7	Day 10	Day 14	Day 17
Wound closure curve	38	38	34	34	30	30
Histology	0	4	0	4	0	4
Total	38	38	34	34	30	30

**Table 5:** Scoring scheme for the different histological parameters to assess wound healing.

Histological parameter	Score					
	0	1	2	3	4	5
Angiogenesis	Absent	Scanty	Low	Moderate	Marked	Profound
Collagen deposition	Absent	Scanty/disorganized	low/fragmented	Moderate/separated	Profound/organized	Restored
Fibroblast infiltration	Absent	Scanty	Low	Moderate	Marked	Profound
Macrophage infiltration	Absent	Scanty	Low	Moderate	Marked	Profound
PMN infiltration	Absent	Scanty	Low	Moderate	Marked	Profound
Fibrin clot	Absent	Scanty	Low	Moderate	Marked	Profound
Epidermal differentiation and indentation	Absent	Scanty	Low	Moderate	Marked	Profound
Multinuclear giant cells	Absent	Scanty	Low	Moderate	Marked	Profound

## 2 Experimental design, materials, and methods

### 2.1 Proteomic analysis

Processed eggshell membrane powder (PEP, <100 μm) [3] was subjected to various extraction and solubilization strategies as utilized previously for ESM proteomics [4](Table 1). A complete protein inventory for PEP was created by merging the proteins identified after application of moderate extraction [lithium dodecyl sulphate/dithiothreitol (LDS/DTT) or urea/ammonium bicarbonate (NH_4_HCO3)] and harsh solubilization conditions [3-mercaptopropionic acid (3-MPA) or sodium hydroxide/dimethylsulfoxide (NaOH/DMSO)] (Fig. 1 and Table 2). Conditions of in-gel (3-MPA, NaOH/DMSO, and LDS/DTT) or in-solution [urea/NH_4_HCO3] tryptic digestion were applied and the resultant peptides were analyzed using the 5600 mass spectrometer with a nanoelectrospray ion source connected to Agilent 1200 nanopump (ES-MS/MS) [3], [4].

### 2.2 Relative abundance of PEP protein constituents

MS/MS peak lists were generated using ProteinPilot (Version 4.5) and analyzed using Mascot (Version 2.4.0) and X!Tandem (CYCLONE version), both programmed to search the TAX_GallusGallus_9031_20141114 database (unknown version, 222,250 entries). Validation of MS/MS based peptide and protein identification was performed using Scaffold Proteome software (version 4.3.4). MS/MS spectra were searched against the Uniprot and NCBI chicken databases. The relative abundance of the PEP protein constituents was estimated by averaging the total spectral count of each identified protein using the aforementioned Scaffold Proteome software (Table 3).

### 2.3 In vivo study

All in vivo experiments were conducted following the approved animal protocol (CMM 2108) by the University of Ottawa Animal Care committee and according to the guidelines of the Canadian Council on Animal Care (CCAC). All animal protocols are in compliance with the NIH Guide for Care and Use of Laboratory Animals (Animal Welfare Assurance # A5043-01). Capacity of PEP for promotion of wound healing was assessed using the well-established mouse excisional wound splinting model [5]and the subsequent macroscopic planimetric timecourse [6] and histological scoring. A total of 38C57BL/6J male mice (10–12 weeks old, Jackson Laboratories, USA) were used for the entire study (Table 4).

### 2.4 Histological assessments

PEP (50 mg) was suspended in 1 mL PBS and centrifuged for 5 minutes at 13,000 rpm. The resultant pellet was centered in a base mould; pre-mounting media composed of 2% agar and 10% formalin was poured gently over the pellet and left for few minutes to solidify. The resulted PEP block was processed using the LOGOS tissue processer, embedded in paraffin and then sectioned using a Leica microtome. PEP sections were stained using Masson trichrome to confirm the absence of stainable collagen in the PEP biomaterial (Fig. 2). For evaluation of wound healing, histological scoring system was established to assess parameters that represent wound healing [7], [8], [9], including degree of angiogenesis, collagen deposition, fibroblast infiltration, macrophage infiltration, polymorphonuclear cells (PMN) infiltration, fibrin clot formation, epidermal differentiation and indentation along with presence of multinucleated giant cells. Every parameter was given a score of 0–5 based on its graded level of abundance. Score 0 indicates complete absence, while score 5 indicates profound manifestation of the assessed parameter. Scoring of collagen deposition was based, not only on the degree of abundance (i.e. absent, scanty, low, moderate, profound, restored), but also on the degree of organization (disorganized, fragmented, separated, organized) (Table 5).
